# Implementation of nasal naloxone across health-care settings: a case report from Ohio

**DOI:** 10.1186/1940-0640-10-S1-A72

**Published:** 2015-02-20

**Authors:** Erin L Winstanley, Angela K Clark, Judith Feinberg

**Affiliations:** 1 Division of Pharmacy Practice and Administrative Sciences, University of Cincinnati College of Pharmacy, Cincinnati, OH, 45267, USA; 2 University of Cincinnati College of Nursing, Cincinnati, OH, 45221, USA; 3 Department of Internal Medicine, University of Cincinnati College of Medicine, Cincinnati, OH, 45267, USA

## 

Drug overdose is now the leading cause of injury death in the United States, surpassing deaths from motor vehicle accidents in 2008 [1]. Naloxone, a short-acting mu opioid receptor antagonist, can reverse an opioid overdose and prevent fatality [2]. Opioid overdose prevention programs (OOPPs) provide education on overdose prevention and some have begun to distribute naloxone. As of June 2010, there were an estimated 188 OOPPs operating in the United States [3]; despite the fact that Ohio has one of the highest overdose fatality rates, there were no OOPPs at that time. The purpose of this study is to: 1) describe overdose prevention education and/or naloxone distribution programs in Ohio across various health-care settings; and 2) identify implementation barriers. A two-page survey was emailed to known Ohio OOPPs and snowball sampling was used to identify other programs. OOPP staff were contacted by phone to confirm their interest in study participation, and 19 of 21 programs completed the survey and are included in this analysis. As of October 2014 these 19 OOPPs were operating in 14 Ohio cities. The primary funding sources were: 1) Ohio Department of Health; 2) Interact for Health (a foundation serving southwest Ohio); and 3) other local public and private agencies. Overall, these programs have distributed 1935 naloxone kits and reported 152 confirmed overdose reversals. There was significant expansion of OOPPs in Ohio after the passage of House Bill 170 in 2014, which removed criminal and civil penalties for clinicians that prescribe naloxone and bystanders that administer naloxone. Identified barriers to OOPPs include: 1) cost in terms of both the provision of naloxone kits and the operational costs associated with prescribing naloxone; 2) stigma of addiction and perceptions of naloxone as a harm reduction strategy that would enable continued drug use; 3) legal/administrative concerns related to possession and distribution of naloxone; and 4) lack of standardized protocols or models that would facilitate operational integration of OOPPs into existing programming. In conclusion, OOPPs have recently expanded significantly in Ohio. Additional research is needed to determine whether this expansion is associated with a decrease in overdose fatalities.
